# An international position statement on practical approaches for inpatient continuous glucose monitoring, insulin pumps and automated insulin delivery systems in adults

**DOI:** 10.1007/s00125-026-06838-8

**Published:** 2026-09-22

**Authors:** Mikkel T. Olsen, Elias K. Spanakis, Hood Thabit, Ketan Dhatariya, Omar G. Mustafa, Parizad Avari, Alistair N. Lumb, Katheleen Dungan, Athena Philis-Tsimikas, Kristen Kulasa, David N. O’Neal, Shashank R. Joshi, Alexandros L. Liarakos, Emma G. Wilmot, Julie L. V. Shaw, Roman Vangoitsenhoven, Chantal Mathieu, Irl B. Hirsch, Priyathama Vellanki, Francisco J. Pasquel, Guillermo Umpierrez, Rodolfo J. Galindo, Ulrik Pedersen-Bjergaard, Peter L. Kristensen, Julia K. Mader, Kirsten Nørgaard

**Affiliations:** 1 Steno Diabetes Center Copenhagen, Herlev, Denmark; 2 Baltimore VA Medical Center, University of Maryland School of Medicine, Baltimore, MD USA; 3 Diabetes, Endocrine & Metabolism Centre, Manchester Royal Infirmary, Manchester University NHS, Manchester, UK; 4 Division of Diabetes, Endocrinology and Gastroenterology, Faculty of Biology, Medicine and Health, University of Manchester, Manchester, UK; 5 Elsie Bertram Diabetes Centre, Norfolk and Norwich University Hospitals NHS Foundation Trust, Norwich, UK; 6 Department of Medicine, Norwich Medical School, University of East Anglia, Norwich, UK; 7 Department of Diabetes, King’s College Hospital, and King’s College London, London, UK; 8 Department of Diabetes and Endocrinology, Imperial College Healthcare NHS Trust, London, UK; 9 Department of Metabolism, Digestion and Reproduction, Imperial College London, London, UK; 10 Oxford Centre for Diabetes, Endocrinology and Metabolism, Churchill Hospital, Oxford, UK; 11 National Institute for Health and Care Research, Oxford Biomedical Research Centre, Oxford, UK; 12 Division of Endocrinology, Diabetes, and Metabolism, The Ohio State University Wexner Medical Center, Columbus, OH USA; 13 Scripps Whittier Diabetes Institute, San Diego, CA USA; 14 Division of Endocrinology and Metabolism, Department of Internal Medicine, University of California, San Diego, La Jolla, CA USA; 15 University of Melbourne Department of Medicine, St. Vincent’s Hospital, Fitzroy, VIC Australia; 16 Lilavati Hospital, Mumbai, Maharashtra India; 17 Department of Diabetes and Endocrinology, University Hospitals of Derby and Burton NHS Foundation Trust, Royal Derby Hospital, Derby, UK; 18 School of Medicine, Faculty of Medicine and Health Sciences, University of Nottingham, Nottingham, UK; 19 Division of Biochemistry, Eastern Ontario Regional Laboratory Association and The Ottawa Hospital, and Department of Pathology and Laboratory Medicine, University of Ottawa, Ottawa, ON Canada; 20 Clinical and Experimental Endocrinology, KU Leuven, Leuven, Belgium; 21 Division of Metabolism, Endocrinology, and Nutrition, Department of Medicine, University of Washington, Seattle, WA USA; 22 Division of Endocrinology, Metabolism and Lipids, Department of Medicine, Emory University School of Medicine, Atlanta, GA USA; 23 Division of Endocrinology, Diabetes and Metabolism, Department of Medicine, University of Miami Miller School of Medicine, Miami, FL USA; 24 Department of Endocrinology and Nephrology, Copenhagen University Hospital – North Zealand, Hilleroed, Denmark; 25 Department of Clinical Medicine, Faculty of Health and Medical Sciences, University of Copenhagen, Copenhagen, Denmark; 26 Division of Endocrinology and Diabetology, Department of Internal Medicine, Medical University of Graz, Graz, Austria

**Keywords:** Automated insulin delivery, Continuous glucose monitoring, Diabetes, Diabetes technology, Hospital, In-hospital, Inpatient, Insulin pump, Position statement

## Abstract

There is an urgent and unmet need for a practical clinical guideline addressing the use of continuous glucose monitoring (CGM), insulin pumps and automated insulin delivery (AID) systems in hospital settings. The goal with this position statement is to facilitate effective and safe use of these devices to improve glycaemic management, enhance outcomes and contribute to standardisation efforts in initiating or continuing the use of these devices in hospitals for non-critically ill hospitalised adults. The topics covered include selection criteria for management with diabetes technologies, CGM-derived glycaemic metrics and goals for adults with diabetes, insulin titration and administration guidelines, perioperative care, CGM alarm settings for hypoglycaemia and hyperglycaemia, clinical staff training and workflows, integration of device data with the electronic health record, imaging tests considerations and discharge planning.

**Graphical Abstract:**

**Figure:**
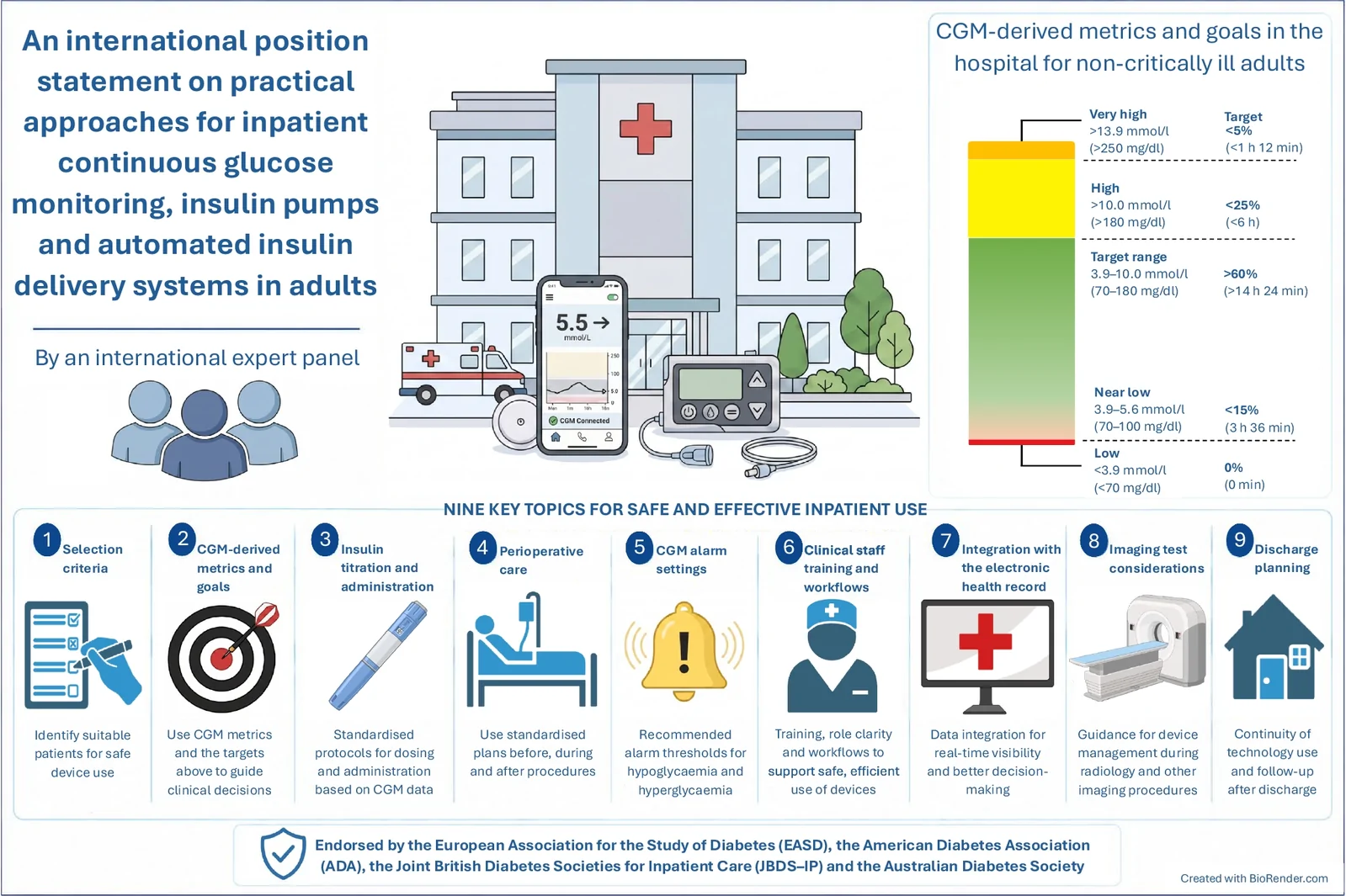

**Supplementary Information:**

The online version contains peer-reviewed but unedited supplementary material available at https://doi.org/10.1007/s00125-026-06838-8.

## Introduction

Continuous glucose monitoring (CGM) has the potential to improve inpatient glucose management by providing real-time glucose levels every one to five minutes, creating a detailed and continuous profile of an individual’s glycaemic status [1–4]. Unlike traditional point-of-care (POC) glucose testing, typically performed three to five times per day in non-critically ill hospitalised individuals, CGM minimises data gaps, which is important in an inpatient setting where high glycaemic variability and rapid shifts in insulin requirements are often seen due to altered glucose metabolism [5]. Frequent glucose readings and real-time alerts for hypoglycaemia and hyperglycaemia enable more precise and responsive insulin titration, which in turn may lead to improved glycaemic status and reduce the risk of complications such as increased morbidity and mortality and prolonged hospital stays [6–10].

While CGM and insulin pumps—the latter which, if integrated with CGM in association with a response algorithm, can work as an automated insulin delivery (AID) system—have greatly improved diabetes management in the outpatient setting, their adoption in hospital settings remains limited [11, 12]. Several barriers hinder harnessing the full potential of these devices in inpatient diabetes care [13, 14]. These barriers include, among others, concerns about accuracy of inpatient CGM, conditions under which use of CGMs, insulin pumps and AID systems is feasible in a hospital setting, limited integration of data into electronic health records (EHR), inadequate clinical staff training on the management of these devices, cost and regulatory concerns, and the absence of standardised guidelines for CGM-guided insulin adjustments in non-AID users [2, 13, 15–19].

## Developing this position statement

An expert panel of 26 healthcare professionals with expertise in inpatient CGM, insulin pumps and AID convened to develop recommendations for the safe and effective use of these technologies in hospital settings for non-critically ill hospitalised individuals. A steering committee (MTO, UP-B, PLK, JKM and KN) drafted key topic areas, which the panel refined through multiple feedback rounds. In assembling the expert panel, the steering committee invited panellists by e-mail. Efforts were made to ensure geographic diversity, representation across different healthcare systems and a balance of gender and professional backgrounds. The panel comprised internationally recognised experts whose experience spans clinical practice, research and guideline development. Participation in the writing group was voluntary, with no financial remuneration or external funding provided.

Nine subtopics were identified through discussion by the entire expert panel and from PubMed searches for articles published up to 10 July 2025, using terms such as ‘continuous glucose monitoring,’ ‘insulin pumps,’ ‘automated insulin delivery systems,’ and ‘inpatient.’ (Full search strings can be found in electronic supplementary material [ESM] 1). Relevant studies identified through these searches, reference lists of retrieved articles and the authors’ personal files were reviewed. Articles in English, German, French, Norwegian, Swedish, Hindi and Danish were included.

In reaching consensus, a previously successful approach was followed [19], in which subgroups within the expert panel reviewed the latest evidence and proposed recommendations for each of the nine identified topics. These were discussed in full-panel online meetings from March to July 2025, with minutes recorded for transparency. Feedback from these meetings informed the final recommendations. Where evidence was not clear, the expert panel discussed the recommendations until consensus was reached.

The final draft was reviewed and approved by all writing group members. Recommendations were graded with the American Diabetes Association (ADA) evidence system (A–C, with expert opinion as E) [20]. An advisory council of six individuals with diabetes provided additional input through separate online meetings, documented in plain language. The work has been endorsed by the Joint British Diabetes Societies for Inpatient Care (JBDS-IP), the Australian Diabetes Society, the European Association for the Study of Diabetes (EASD) and ADA. For all of the following, recommendations are based on literature and clinical experience in adults and should not be directly extrapolated to paediatric populations.

## 1. Selection criteria for management with CGM, non-AID insulin pumps and AID in hospitalised adults

### Evidence about inpatient use of CGM

Randomised controlled trials (RCTs) confirm that CGM reduces hypoglycaemia as compared with POC testing in hospitalised individuals with type 1 or type 2 diabetes treated with basal-bolus therapy [1–3]. Hypoglycaemia is linked to longer stays and higher mortality [21]. Guidelines support both continuation of individuals’ own devices during hospitalisation and initiation of new CGMs at admission [22–24]. A meta-analysis of six RCTs (979 participants with diabetes) in non-intensive care unit settings found that in-hospital CGM use improved time spent with glucose in target range (TIR) 3.9–10.0 mmol/l (70–180 mg/dl) by about 7 percentage points (%-points) in comparison with POC glucose testing, despite significant variability among trials [4]. More recent RCTs not included in the meta-analysis—the Time in Glucose Hospital Target (TIGHT) trial [25] and a trial by Thabit et al [26]—found no significant glycaemic benefit of CGM, whereas the Diabetes Team and CGM in Managing Hospitalised Patients With Diabetes (DIATEC) trial showed that CGM combined with an insulin titration protocol and inpatient diabetes team increased TIR by about 15%-points and reduced hypoglycaemia, hyperglycaemia, glycaemic variability, insulin use and complications and improved treatment satisfaction in hospitalised individuals with type 2 diabetes [1, 27]. Systematic reviews have previously been published summarising the trial characteristics of the above-mentioned RCTs investigating CGM vs POC testing in individuals with type 1 and type 2 diabetes [4, 28].

Differences in glycaemic and clinical outcomes across studies likely reflect absence of CGM-based insulin titration protocols (section 3), variations in staff training (section 6) [16, 28, 29] and uncertainty regarding which inpatient subgroups benefit most. A heterogeneity of treatment effect analysis suggested that, in the population investigated, all non-critically ill hospitalised individuals with type 2 diabetes treated with basal-bolus therapy during hospitalisation benefit from CGM over POC testing on glycaemic outcomes, regardless of age, sex, HbA_1c_, BMI, diabetes duration, admission glucose levels, admission diagnosis, outpatient treatment plans and other characteristics [30].

### Evidence about inpatient use of non-AID insulin pumps and AID

Few clinical trials are available for the inpatient initiation or continuation of non-AID insulin pumps or AID systems; however, available studies have demonstrated that these devices improve glycaemic outcomes.

Gu et al reported that in an RCT of 81 hospitalised individuals with type 2 diabetes, non-AID insulin pump therapy with CGM reduced hypoglycaemia and hyperglycaemia in comparison with multiple daily injection (MDI) and POC testing [31]. Single-arm trials have recently been published showing the feasibility of implementing inpatient AID [32, 33] as well as the benefits of continuing non-AID insulin pumps or AID during hospitalisation in non-critically ill hospitalised individuals with type 1 and type 2 diabetes [34, 35]. Additionally, two RCTs have evaluated the efficacy of inpatient AID in individuals with type 2 diabetes compared with that of MDI and continuation of individuals’ outpatient glucose-lowering therapies [36, 37]. A third RCT, with hospitalised individuals with type 2 diabetes and pancreatogenic diabetes, included similar comparisons [38]. Across all three trials, AID resulted in a 20–30%-point increase in TIR 5.6–10.0 mmol/l (100–180 mg/dl). AID systems have been found safe for use intrapartum and postpartum in pregnancies complicated by type 1 diabetes, as recently reviewed, although few trials are available [39]. Implementation challenges, including workflow integration and cost-effectiveness, remain insufficiently studied [33, 40]. Nonetheless, discontinuing hospitalised individuals’ use of self-managed non-AID pump or AID systems may cause frustration and a perceived loss of control [41].

### Risk factors for inpatient hypoglycaemia and hyperglycaemia

Pending further evidence, the expert panel recommends prioritising CGM, non-AID insulin pump or AID for non-critically ill hospitalised individuals at high risk of hypoglycaemia [42–45] or hyperglycaemia [42, 46–49] with final decisions guided by individual clinical judgement (Table 1). Risk factors largely overlap, potentially due to the common denominator of high glycaemic variability.

**Table 1:** Risk factors for hypoglycaemia and hyperglycaemia in non-critically ill hospitalised individuals with diabetes

Risk factors for hypoglycaemia	Risk factors for both hypoglycaemia and hyperglycaemia	Risk factors for hyperglycaemia
• Chronic liver disease or liver failure/cirrhosis • History of cerebrovascular accident • Aggressive insulin treatment plans during hospitalisation • Mismatch of POC glucose testing with insulin dosing and meal delivery • Frequent insulin dosing • Failure to address changes in glucocorticoid doses or nutritional therapies • Impaired hypoglycaemia awareness • History of severe hypoglycaemia • Poor meal intake • Nonstandard therapies: sulfonylurea, premixed insulins and concentrated insulins (e.g. U-500 regular insulin and U-300 glargine) • Correctional insulin overnight	• Advanced age • Acute and chronic kidney injury • Long duration of diabetes • Multiple and severe comorbidities (e.g. as assessed with the Charlson comorbidity index [103]) • Low BMI • Low C-peptide	• Insulin treatment before hospitalisation • High HbA_1c_ levels at admission • Male sex • Elevated LDL-cholesterol levels • Enteral or parenteral nutritional therapies • Glucocorticoid therapies

#### Recommendations

*Initiating diabetes device(s) in device-naive hospitalised individuals*

- Initiating CGM in device-naive hospitalised individuals is feasible, especially when done in consultation with inpatient diabetes teams or on diabetes specialist advice [28]. **B**
- Initiating non-AID insulin pumps or AID systems in hospitalised individuals who are not already equipped with these devices may be more challenging [50], even though the indications for CGM, as outlined below, may also apply to these devices. This initiation should be done in consultation with inpatient diabetes teams or on diabetes specialist advice. **E**

*Continuation of an individual’s own diabetes device*

- If the hospitalised individual or the clinical staff can safely support the use of CGM, CGM should be continued [1, 4, 25–27]. **B**
- If the hospitalised individual can safely manage the non-AID insulin pump or AID system, it should be continued. Given rapidly changing clinical conditions, self-management ability should be reassessed at each contact [41]. **C**
- Hospitalised individuals are strongly encouraged to share glucose and/or insulin data with the clinical staff. Those using CGMs should be informed that the hospital’s POC blood glucose meter will still be used alongside CGM to provide validation for CGM readings when indicated (section 6). **E**

*Situations for considering CGM use to support hypoglycaemia prevention*

- Hospitalised individuals with diabetes (any type) who were treated with insulin or other hypoglycaemia-inducing medications prior to admission, initiated insulin therapy during hospitalisation, have a history of frequent hypoglycaemic events or experienced hypoglycaemia during the current hospital stay [42–45].** B**
- Hospitalised individuals considered at high risk of hypoglycaemia due to hypoglycaemia unawareness or one or more risk factors (Table 1) [42–45]. **B**
- Cognitive impairment (if clinical staff can manage CGM use and have access to glucose data). **E**

*Situations for considering CGM use to support hyperglycaemia prevention*

- Hospitalised individuals with diabetes treated with insulin prior to admission [42, 46–48]. **C**
- Hospitalised individuals with diabetes and multiple severe comorbidities [42, 46–48]. **C**
- Hospitalised individuals with a high HbA_1c_ >75 mmol/mol (>9%) [51]. **C**
- Hospitalised individuals with preexisting diabetes treated continuously with systemic glucocorticoids in moderate-to-high doses, >10 mg/day (prednisolone equivalents) and with glucose levels >10.0 mmol/l (>180 mg/dl) for repeated glucose tests. Otherwise, glucocorticoids >30 mg/day (prednisolone equivalents) in the case of new-onset hyperglycaemia without preexisting diabetes and with glucose levels >10.0 mmol/l (>180 mg/dl) for repeated glucose tests. **E**
- Hospitalised individuals with diabetes who are treated with enteral or parenteral nutrition therapies. **E**

*Contraindications to (continuation of) CGM, non-AID insulin pump or AID system*

- Inability of the hospitalised individual or the clinical staff to manage the devices. **E**
- Impaired level of consciousness (excluding anaesthesia) interfering with the individual’s ability to engage in diabetes care. (CGM may be continued if clinical staff can manage it and have access to glucose data.) **E**
- Diabetic ketoacidosis or hyperosmolar hyperglycaemic state [52]. **C**
- Insufficient supply of CGM or pump materials during hospitalisation. **E**
- Specific imaging procedures (section 8). **B**
- Medications or conditions interfering with CGM accuracy (section 6). **B**

## 2. CGM-based glycaemic metrics and optimal goals for adults with diabetes

Several glycaemic metrics have been proposed for inpatient research [19], but for clinical practice, to guide decision-making, a simpler set of core measures is needed. Unlike in the ambulatory setting, where CGM data from at least 14 days with >70% sensor use is the recommendation, hospitalised individuals are typically discharged before this amount of data can be collected. Moreover, inpatient diabetes management requires a more day-to-day approach due to rapidly changing glucose metabolism and oral intake.

**Fig. 1:**
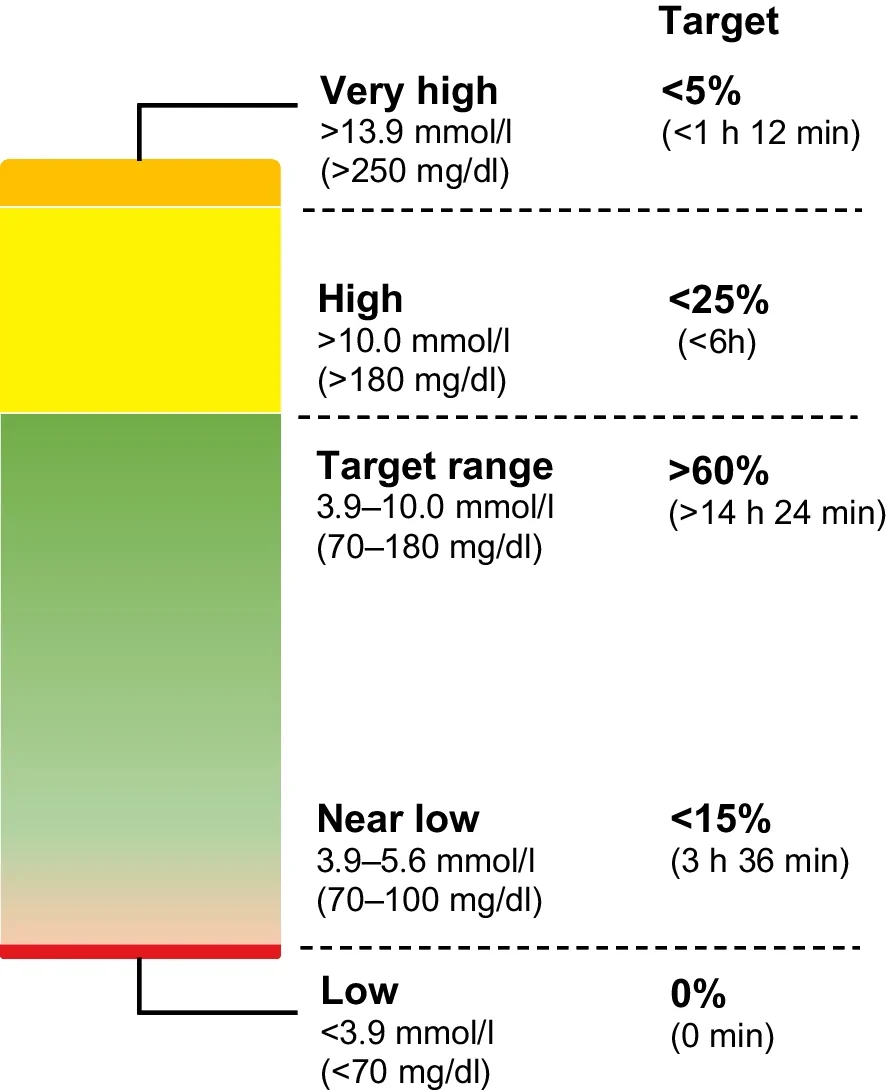
Recommendations for CGM-based metrics and goals for non-critically ill adult hospitalised individuals with diabetes (all types)

While uncertainty remains as to the relationship between CGM-derived metrics and hospital outcomes, evaluating daily time spent with glucose in different ranges provides a practical framework for inpatient use. Unlike with outpatient goals, with a focus on preventing long-term risks [53], inpatient glycaemic goals should prioritise avoidance of hypoglycaemia, prevention of acute complications and optimisation of overall outcomes. The recommended goals outlined below represent minimum requirements during the entire hospitalisation for individuals using CGM with or without integration with other diabetes devices (Fig. 1).

### Recommendations

- TIR 3.9–10.0 mmol/l (70–180 mg/dl) is recommended to be above 60%. **E**
- TAR >10.0 mmol/l (>180 mg/dl) is recommended to be below 25%. **E**
- TAR >13.9 mmol/l (>250 mg/dl) is recommended to be below 5%. **E**
- As hypoglycaemia is the main concern in the hospital setting, TBR <3.9 mmol/l (<70 mg/dl) is recommended to be 0%. **E**
- Time near low (TNL) 3.9–5.6 mmol/l (70–100 mg/dl) should be kept below 15% to reduce the risk of impending hypoglycaemia [54, 55]. **C**
- Individualised glycaemic goals, tailored to individual needs, may help to reduce the risk of both hyperglycaemia and hypoglycaemia, associated with adverse hospital outcomes. **E**

## 3. Insulin titration and administration based on CGM in hospitalised adults on MDI, non-AID insulin pump or AID

### 3.1 CGM-based insulin titration for hospitalised adults on MDI

**Fig. 2:**
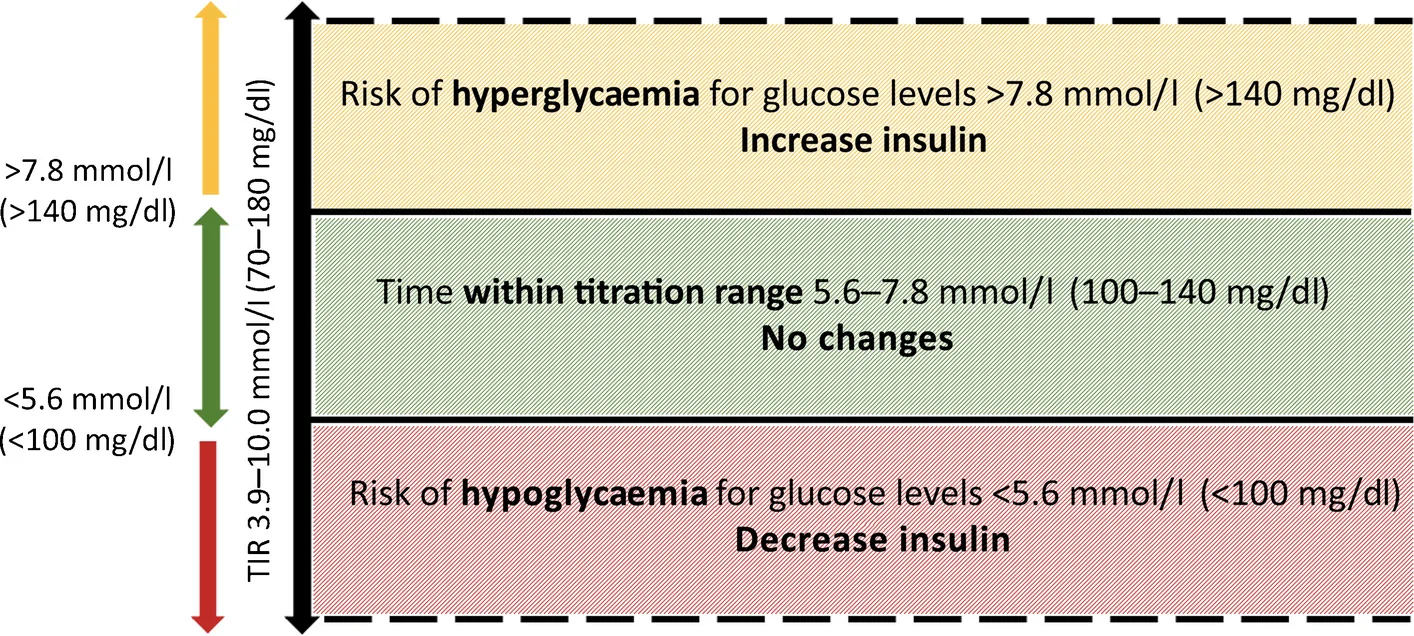
Three glycaemic ranges to consider in aiming for an overall TIR of 3.9–10.0 mmol/l (70–180 mg/dl) during hospitalisation. For the recommendations of increasing insulin (yellow area) or decreasing insulin (red area), the proposed CGM-based insulin titration protocol (ESM 2) may be used as a guide

Historically, concerns about overlapping insulin doses (‘insulin stacking’) and hypoglycaemia have limited use of CGMs in hospitals [17]. Experts in inpatient diabetes care have advocated for CGM-based insulin titration protocols; however, consensus is lacking [19, 22, 23]. Compared with POC glucose testing, CGM provides continuous insight into glucose trends within and outside the target range (Fig. 2). Yet, the abundance of data may also create uncertainty in clinical decisions and contribute to therapeutic inertia when standardised CGM-based insulin titration protocols are absent [56].

A recent systematic review of nine RCTs found that the best glycaemic outcomes were achieved with those CGM-based insulin titration protocols where detailed, structured guidance was provided and diabetes-trained staff were involved [28, 56]. Currently, only one validated CGM-based insulin titration protocol offers comprehensive guidance for CGM use in hospitalised individuals with type 2 diabetes with demonstrated superior inpatient glycaemic and clinical results, forming the basis of the proposed protocol (ESM 2) [56, 57].

### 3.2 Non-AID insulin pump and AID users

In-hospital glucose management for insulin pump users depends on whether insulin delivery is automated or not. In AID systems, insulin delivery is continuously adjusted on the basis of real-time CGM data and typically no external modification is needed, except for meal-related boluses (Table 2). Supplemental insulin should be administered through the pump for both AID and non-AID users.

**Table 2:** Insulin treatment from non-AID insulin pump and AID systems

Non-perioperative care (applies also to hospitalised non-surgical individuals) • Non-AID insulin pump: Insulin titration for basal rate insulin and prandial insulin may follow the same adjustments as for an MDI plan in ESM 2 (where basal insulin in the MDI plan corresponds to basal rate for non-AID insulin pump users). • AID systems: Automated mode should be continued. In hospitalised individuals who are not eating or at risk for hypoglycaemia (section 1), raising the target or use of nonaggressive insulin titration modes (e.g. exercise mode) may be feasible. ‘Sleep activity’, offered by some AID systems to maintain a narrower overnight glucose target and to pause automated correction boluses during this period, can be used when possible to reduce nocturnal hypoglycaemia. • Both non-AID insulin pump and AID systems: Transitioning to an MDI plan (Table 3) should only be considered if there are contraindications to continuing the use of the CGM, non-AID or AID system (section 1) or if the insulin requirements increase to a level where the AID system can no longer maintain adequate glycaemic management. In such instances, continuing the AID system in non-automated mode may be a viable alternative.
Perioperative care • Non-AID insulin pump: Adjust the basal rate hourly during intraoperative care or when clinically indicated. Insulin titration for basal rate insulin and prandial insulin may follow the same adjustments as for an MDI plan in ESM 2 (where basal insulin in the MDI plan corresponds to basal rate for non-AID insulin pump users). • AID systems: Same as for non-perioperative care. • Both non-AID insulin pump and AID systems: The individual should bring all necessary pump supplies (if not hospital provided) to the hospital and insert a new subcutaneous infusion set outside the planned surgical area the day before surgery. Before the surgical procedure, a qualified healthcare professional should ideally assess and, if necessary, adjust the patient’s insulin pump settings. It should be confirmed that there is enough insulin in the pump’s reservoir to prevent it from running out on the day of the surgery. During the surgical procedure, the anaesthesiologist must have access to the insulin pump, allowing for suspension or disconnection if necessary. In the event of abrupt discontinuation, management in hospitalised individuals should be by insulin infusion corresponding to the basal rate as per local guideline.

For non-AID pump users, inpatient insulin titration may be needed due to fluctuating insulin requirements, but there are no validated guidelines. Basal rates and prandial doses should therefore be adjusted at clinical discretion or according to proposed recommendations (Table 2). If pump discontinuation is required, standard protocols should be used for transition to MDI or intravenous insulin (Table 3).

**Table 3:** Scheduled transition from non-AID insulin pump or AID to pen-based insulin plan for non-perioperative care (applies also to hospitalised non-surgical individuals)

Insulin management • Determine the 24 h basal dose of insulin from the pump settings and give basal insulin subcutaneously 2 h before discontinuing insulin pump therapy. Of note, some AID systems, such as Omnipod 5, may not offer detailed information on basal patterns and instead report only the total daily insulin dose. In these cases, the total daily dose can be divided evenly between basal and prandial insulin. Alternatively, the basal and prandial components can be estimated with the formulas in ESM 2. • Correctional insulin with rapid-acting insulin is to be added when hyperglycaemia is observed, according to the usual institutional guideline or an individual’s known insulin sensitivity (correction) factor. • Adjust basal and prandial insulin doses daily from CGM-based glucose levels (ESM 2).
Resuming non-AID insulin pump or AID • The non-AID insulin pump or AID treatment can be resumed when no contraindications are present anymore (section 1). • For non-AID insulin pump therapy, the pump can be resumed with the normal basal insulin rate 22 h after the last basal insulin injection by pen. • AID systems can be resumed once the effect of the injected basal insulin has substantially worn off (e.g. 22 h after last injection) to prevent underestimation of basal and correction needs the following day.

### Recommendations

- For insulin titration and administration using CGM and an MDI plan, a standardised CGM-based insulin protocol (e.g. ESM 2) should be followed [28]. **A**
- CGM systems used for insulin dosing should meet integrated CGM (iCGM) criteria required by the US Food and Drug Administration (FDA) [58]. **E**
- Use of non-AID insulin pumps should be supported by frequent clinical assessments to guide insulin titration (Table 2) [31–35, 41]. **C**
- AID systems should operate with minimal user input, except around mealtimes when manual input is required (Table 2) [36–39, 41]. **C**
- When carbohydrate counting is impractical, prandial insulin may be estimated as follows: 40% of the total daily insulin dose (if the total daily insulin dose is known, otherwise according to ESM 2), divided among meals if intake is adequate. Individuals with minimal oral intake may need little or no prandial insulin, while those on glucocorticoids, nutritional support or experiencing stress hyperglycaemia may require a higher proportion (50–60%) of total daily dose as prandial insulin [59]. **C**
- Management in individuals in the case of nil per os (NPO) should be with insulin infusion per local guidelines or with continuation of a non-AID pump with a reduced basal rate (10–25%) or an AID system, with glucose- or dextrose-containing intravenous fluids. Transition to enteral or parenteral nutrition with NPO for >1–2 days or as per local guideline. **E**
- Hospitals should establish policies that define responsibilities for the continuation of CGMs, non-AID insulin pumps and AID systems. **E**

## 4. Perioperative care with CGM, non-AID insulin pump or AID in hospitalised adults

Differentiating between perioperative and non-perioperative care is clinically relevant for hospitalised individuals with diabetes because surgery-related stress, fasting, anaesthesia and rapid changes in insulin sensitivity substantially affect glycaemic management. A recent systematic review of 26 studies involving 1016 hospitalised individuals assessed the use of CGM vs POC glucose testing perioperatively. However, due to the inclusion of only two RCTs, the benefits of CGM on perioperative glycaemic management and clinical outcomes remain uncertain [60]. Device malfunctions (typically accidental removal and sensor issues) were observed in 9% of cases during perioperative care, primarily in the pre- and postoperative phases, while CGM use during surgery remains less studied. In a more recent RCT in individuals hospitalised for orthopaedic surgery, CGM increased TIR 3.9–10.0 mmol/l (70–180 mg/dl) by 15%-points in the pre- and postoperative periods, along with fewer hospital complications, compared with POC glucose testing [1].

Clinical recommendations suggest continuing non-AID insulin pump therapy during the perioperative period [61–65]. Recent studies support the safe use of CGMs and non-AID insulin pumps for hospitalised individuals with diabetes undergoing shorter surgical procedures, showing improved glycaemic outcomes and reduced hypoglycaemia [66–70], particularly when use is aligned with institutional guidelines for perioperative management [71, 72].

The recommendations and evidence for AID systems are limited [73]. A study by Herzig et al [74] enrolled 45 adult surgical hospitalised individuals with diabetes (other than type 1) undergoing surgeries lasting more than 2 h. These hospitalised individuals were assigned to either AID or usual care. The study found no adverse events and an improvement in TIR of 5.6–10.0 mmol/l (100–180 mg/dl) with AID. These findings suggest that with optimal guidelines and familiarity with non-AID insulin pump and AID systems, such technologies can be safely integrated into perioperative care.

### Recommendations

- CGM and CGM-based insulin dosing can be safely continued in perioperative care when appropriate [1, 60]. **B**
- Perioperative care for short surgical procedures (no more than one missed meal) allows for continuation of the non-AID insulin pump or AID. For longer or more complex surgical procedures with a high risk of prolonged surgery time, non-AID insulin pump and AID users should be switched to intravenous insulin infusion (Table 2) [1, 60, 66–72, 74]. **C**

## 5. CGM alarms for hypoglycaemia and hyperglycaemia in hospitalised adults

The use of CGM alarms for low or high glucose levels in hospitals is recommended to help prevent dysglycaemia while minimising the risk of alarm fatigue [23, 75]. While some alarms may be customised, others, such as those for urgent low glucose, must remain active and cannot be adjusted. Setup for telemetric viewing of the alarms and real-time CGM-based glucose levels for clinical staff have been described previously [1, 76]. Alternatively, cognitively capable hospitalised individuals may alert medical staff when alarms are triggered.

Hyperglycaemic alarm settings have often been used as safety measures, typically set at 13.9 mmol/l (250 mg/dl) to prevent severe hyperglycaemia [2, 26, 51, 56] or at individual-specific thresholds, which may effectively individualise diabetes care for low- and high-risk hospitalised individuals and reduce alarm fatigue [26]. A greater discrepancy in hypoglycaemic alarm settings has been observed in inpatient trials using CGM. In some trials the alarm threshold has been set at 3.9 mmol/l (70 mg/dl), corresponding to the entry level of hypoglycaemia [26, 56, 77]. In other trials more precautionary thresholds were used to avoid hypoglycaemia altogether, i.e. settings at 4.7 mmol/l (85 mg/dl) [3] and 5.0 mmol/l (90 mg/dl) [51]. More data are needed before implementation of CGM trend arrows or predictive alarm functions in inpatient diabetes care.

### Recommendations

- Hypoglycaemic alarms should be set at 4.4 mmol/l (80 mg/dl) (alerting immediately) for most hospitalised individuals or at a higher threshold for hospitalised individuals at high risk of hypoglycaemia (section 1). Hypoglycaemia should be treated according to the usual institutional guideline [28, 56, 77]. **C**
- Hyperglycaemic alarm thresholds should be set at 13.9 mmol/l (250 mg/dl), triggering alerts immediately, or after a customisable delay, a feature offered by some CGM systems. Alarm settings may be lower for hospitalised individuals with well-regulated diabetes or those with type 1 diabetes or insulinopenic type 2 diabetes and higher for hospitalised individuals with dysregulated diabetes to reduce the risk of frequent alerts leading to alarm fatigue [28]. **C**
- For avoidance of insulin stacking and the risk of later-onset hypoglycaemia, rapid-acting correctional insulin should only be administered if more than 3 h have passed since the previous rapid-acting insulin dose (>4 h for regular human insulin), regardless of whether the dose is given in response to a hyperglycaemia alarm or as part of scheduled correctional insulin administration [1, 29]. **E**

## 6. Clinical staff training and workflows around CGM, non-AID insulin pumps and AID in hospitalised adults

Experts suggest that sufficiently trained clinical staff, such as in-hospital diabetes teams with advanced knowledge of CGM, will be important in implementing CGMs in the hospital [2, 15–19, 78]. There is little evidence on this for non-AID insulin pumps and AID systems, but the same arguments apply.

Clinical staff must be aware that interference with CGMs by substances, such as medications, can result in inaccurate glucose readings, either too high or too low. The extent of this interference depends on the concentration of the substance and on the device itself. Many medications used in hospitals have not been tested for potential impact on CGM accuracy, and their effects remain unknown. Known interferences vary across CGM brands and some common medication interferences are as follows [19].

- Acetaminophen (in doses higher than recommended)
- Ascorbic acid
- Hydroxyurea
- Other reducing agents/compounds depending on the device

In addition, inaccurate readings can occur due to poor peripheral blood circulation, hypoglycaemia, severe hyperglycaemia, compression of the device, acidosis and severe anaemia [79–81].

### Recommendations

**Fig. 3:**
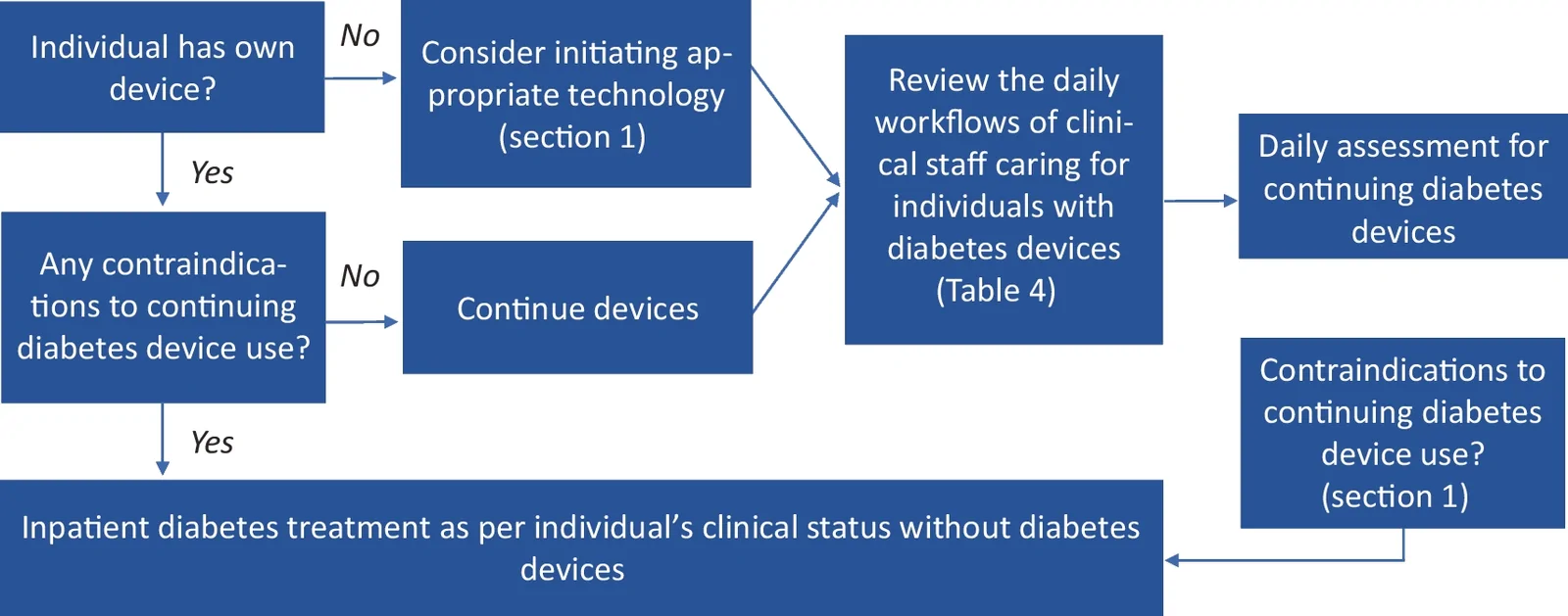
Workflow to guide clinical staff in managing hospitalised individuals with diabetes using diabetes devices

- The expert panel recommend clinical staff training concerning inpatient CGM, non-AID insulin pumps and AID systems (Table 4) [28]. **C**
- Clinical staff must evaluate the feasibility and safety of continuing or initiating use of diabetes devices (Fig. 3). **E**
- Confirmatory POC glucose testing should be performed during the first 24 h after insertion of the CGM, e.g. before meals (or every 4–6 h for NPO), before bedtime and at night and/or where accuracy concerns arise, such as in the case of hypoglycaemia <3.9 mmol/l (<70 mg/dl) or hyperglycaemia >15.0 mmol/l (>270 mg/dl), when the sensor reading is discordant with clinical symptoms or condition or when interfering substances are present [80]. **B**
- Calibration of the CGM (if possible) with POC glucose tests should be performed when the difference exceeds 20% for CGM values >5.6 mmol/l (>100 mg/dl) or exceeds ±1.1 mmol/l (±20 mg/dl) for CGM values <5.6 mmol/l (<100 mg/dl) [82, 83], and at stable glucose concentrations [23, 84]. **C**
- Persistent discrepancies (as defined above) between CGM and POC glucose testing warrant CGM replacement or discontinuation. **E**
- In the case of discrepancies between POC and CGM readings, the lower reading should be used to guide clinical decision-making, to minimise the risk of hypoglycaemia. **E**

**Table 4:** Workflows and good practice points for inpatient CGM, non-AID insulin pumps and AID systems for clinical staff

Setting up and maintaining the devices • Safe insertion and removal of the CGM system, non-AID insulin pump or AID, including manufacturer-recommended sensor insertion site. • Preferably, transmitting CGM signals to the nursing stations or elsewhere for telemetric viewing. • When and how to remove the CGM and/or non-AID insulin pump or AID, e.g. due to run-out time, contraindications (section 1) or imaging procedures (section 8). • Be aware of performing POC glucose tests in the sensor warm-up period and the first 24 h after insertion (section 6). • When to perform a POC glucose test with a standard glucometer to validate CGM accuracy (section 6). • Consider protective covers to prevent dislodgement or pressure-induced false lows (CGMs only), particularly in the case of frequent repositioning and during transfers and periods of prolonged bed rest for the individual. • Pumps only: include a contingency plan detailing the actions to be taken if discontinuation of the non-AID insulin pump or AID system is needed unexpectedly, such as due to an extended surgical procedure or contraindications (section 1). • Pumps only: ensure correct infusion set priming. The infusion site should be monitored for any signs of inflammation, leakage or other issues.
Insulin titration for CGM users and insulin pump users • Sessions on how to use the CGM-based insulin titration protocol (ESM 2) and CGM visualisation tools (e.g. via the CGM manufacturer’s official software or in telemetric setups). • Interpreting trends over time in CGM glucose levels rather than single data points as with traditional POC glucose testing. • Training in avoiding insulin stacking by only reacting to hyperglycaemic alarms and not to random viewing of the CGM glucose levels.
CGM alarms • Appropriate settings for hypoglycaemia and hyperglycaemic alarms (section 5). • Appropriate actions taken in response to alarms, including response time (section 5).
Documenting in the EHR • Instructions on how and why CGM data, non-AID insulin pump data or AID data documentation is important in the EHR (Table 5).

## 7. Integration of CGM, non-AID insulin pump, and AID data with the EHR for hospitalised adults

Integrating data from CGM, non-AID insulin pumps or AID systems into the EHR presents several technical challenges [29, 85]. Current EHRs are typically designed to store intermittent clinical measurements, not continuous streams of biometric data, so the direct inclusion of CGM and insulin pump data is challenging.

While intermittent documentation of summary reports and historical CGM metrics is essential for follow-up in the ambulatory setting, the major potential for CGM in hospitalised individuals requires real-time data. Integration of such data into EHRs could function similarly to integration in cardiac telemetry [76], although based on glucose readings and alerts for hypoglycaemia or hyperglycaemia as proposed in the Integration of Continuous Glucose Monitor Data into the Electronic Health Record (iCoDE) project [86–88].

The iCoDE authors recommend standardised and user-friendly CGM reports alongside insulin dosing information. These reports must be compatible with multiple EHR systems and easily accessible to all healthcare professionals. Also, multiple different CGM, non-AID insulin pump and AID systems must be integrated to allow for both initiation of new devices during hospitalisation and the continuation of an individual’s own device with integration of data in the EHR with the hospitalised individual’s permission. Currently, in the outpatient setting, several manufacturers support EHR integration, using a third-party vendor, that ensures consistency and interoperability, and these CGM integrations also comply with privacy and data protection standards [89].

A second challenge, the large data volume from CGM and insulin pump devices, could strain network and server capacity. iCoDE favours a ‘data pull’ into the EHR for outpatient care. However, for inpatient real-time monitoring, a temporary ‘data push’ may be needed, which would require a standardised data structure or use of a third-party data lake accessible to the EHR to manage higher data flow.

Third, when CGM is used in the hospital for real-time glucose monitoring, legal considerations arise regarding accountability for data oversight and clinical response. These considerations also extend into potential liability in the event of technical failures affecting data completeness, timeliness or validity, such as signal loss, lag time or missed alarms. For a successful data pull and linkage, enough patient identifiers must match between the CGM system/portal and EHR.

### Recommendations

- Until an integration between the EHR, CGM, non-AID insulin pump and AID is available, clinical staff must manually document relevant information regarding these devices in the EHR if clinical decisions are based on these devices. In addition, consideration should be given to including the (summary) report for the inpatient period in the EHR on discharge (Table 5). **E**
- Clinicians may benefit from development of standard phrases and local hospital policies to ensure timely and correct documentation in the EHR. **E**

**Table 5:** Points to consider in documenting CGM, non-AID insulin pump or AID data in the EHR

Setting up and maintaining the devices • Document the type of CGM, non-AID insulin pump or AID. Note whether the device is handled by the individual or use is dependent on staff supervision, and whether the pump is in manual mode or AID mode. • Document the location/insertion site of the CGM, non-AID insulin pump, or AID and when replacement should occur (e.g. due to run-out time).
Registration of CGM, non-AID insulin pump or AID data • Glucose levels from CGM should be clearly distinguishable from POC glucose measurements obtained with standard glucometers or laboratory tests whether data are registered as CGM summary statistics/metrics or individual glucose measurements. • All individual glucose levels or CGM-based summary statistics/metrics should be documented if deemed important for clinical decision-making. • The ability of hospitalised individuals to self-manage CGM, insulin pumps or AID should be assessed daily and, if inadequate, and if clinical staff cannot support device use, the therapy should be reevaluated (section 1). • Pumps only: document the individual’s insulin pump settings including glycaemic goals and current basal rate (non-AID insulin pump users only) and estimated basal insulin delivery (AID users).
Insulin titration • Document how basal, prandial and/or correctional insulin was titrated by clinical discretion or by a standardised CGM-based insulin titration protocol (ESM 2). • Plan for how often insulin titration should be done, e.g. daily or at clinical discretion.
CGM alarms • Settings for hypoglycaemia and hyperglycaemic alarms including response time (section 5). • Actions taken, including response time.

## 8. Imaging tests with CGM, insulin pump or AID in hospitalised adults

Evidence is emerging that keeping the CGM on during x-rays or computed tomography (CT) procedures is safe both during and after the procedure [90–93], while evidence is sparser for MRI [92]. It is essential to recognise that temporarily discontinuing the use of these devices could pose a higher risk than keeping them on during imaging or radiation therapy [94]. Recently, the FreeStyle Libre 2 and 3 systems (Abbott, Chicago, IL) were cleared by FDA for safe use after x-rays, CT scans and MRI and therefore do not need to be removed before imaging [95]. CGM readings may be temporarily affected during an MRI, but functionality fully returns to normal after 1 h [96]. The same applies to diathermy, but there is little evidence [81]. The Eversense CGM (Senseonics, Germantown, MD) is cleared for x-rays, CT scans and MRIs when the transmitter is removed [97]. To date, no clinically relevant interference between CGM systems and implantable cardioverter-defibrillators, pacemakers or electrosurgical instruments (e.g. electric scalpels) has been reported in the literature [98]. Nevertheless, caution is advised [81].

For hospitalised individuals using non-AID insulin pumps and AID systems, it is recommended that both the pump and the metal cannula be removed before undergoing MRI [11]. Additionally, it has been suggested that the pump be covered with a lead apron during x-rays or CT scans [11].

### Recommendations

- The expert panel recommends adhering to the manufacturer’s guidelines for the specific device in question to ensure safe management during imaging procedures. **E** The FDA endorses following the manufacturer’s guidelines when it is both safe and feasible to do so [94].

## 9. Discharge planning after being managed with CGM, non-AID insulin pump or AID in hospitalised adults

When the discharge plan involves adjustments to the individual’s usual diabetes treatment, several factors should be considered, including the individual’s available resources, the presence of cardio-renal comorbidities, the individual’s response to medication during hospitalisation, and HbA_1c_ levels. In most cases, an HbA_1c_ level of <53 mmol/mol (<7%) is considered the target for effective diabetes management, where achievable without significant risk of hypoglycaemia [99]. The benefit of adding CGM use is that an at-target HbA_1c_ level can be balanced against the risk of hypoglycaemia and high glycaemic variability.

Use of CGM prior to hospital discharge may reduce readmission rates and emergency department visits [100]. A recent pilot RCT in people with insulin-treated type 2 diabetes found that, compared with POC glucose testing, CGM use after discharge was associated with improved glycaemia and decreased glycaemic variability [101]. Also, a pooled analysis of two RCTs demonstrated that CGM was superior to POC glucose testing in assessment of glucose levels in older adults, with higher detection rates for hypoglycaemic episodes following hospital discharge [102].

### Recommendations

- CGM-detected hypoglycaemia and hyperglycaemia should be addressed in the discharge plan. Follow-up care in the outpatient clinic may be needed. **E**
- Hospitalised individuals with suboptimal glucose levels during hospitalisation and confirmed dysglycaemia at outpatient follow-up may be considered for routine CGM use as part of ongoing outpatient care. This decision should be guided by local guidelines, reimbursement policies and a shared decision-making process with the person living with diabetes. The CGM discharge plan should include a follow-up outpatient appointment within approximately 2 weeks and ensure that the CGM device is connected to the outpatient practice for review at that visit. **E**
- Adjustments to non-AID insulin pump or AID system settings should ideally be made in the outpatient setting, once the effects of the acute illness have resolved and usual activities have been resumed. **E**

## Future perspectives

It is essential to evaluate the implementation of CGM, non-AID insulin pump and AID systems in real-world inpatient diabetes care, outside of strictly controlled research environments. Further research is needed to explore and evaluate use of trend arrows from CGMs in inpatient settings to guide clinical decisions, as CGM trend arrows may help with prevention of impending hypoglycaemia and hyperglycaemia. In 2020, the FDA issued a non-objection to use of CGM in the hospital setting. However, formal regulatory approval for its use during hospitalisation is still pending from the FDA in the USA, from the European Commission, responsible for Conformité Européenne (CE) marking in accordance with the Medical Device Regulation (MDR 2017/745) in the European Union and for other regions.

Gathering sufficient data on whether different CGM systems are accurate enough for standalone use, or whether adjunctive use of POC glucose tests is necessary, remains a priority. Additionally, there is limited evidence on the effects of continuing use of an individual’s own CGM during hospitalisation. There is a significant gap in data regarding the effectiveness and safety of non-AID and AID systems in the inpatient setting. Moreover, cost–benefit analyses of diabetes technology use in hospitals are still limited, along with data on individual and healthcare professional satisfaction with these technologies in the inpatient setting.

Future research should examine whether these technologies perform differently across distinct groups—including people with type 1 diabetes, those with type 2 diabetes and individuals who are either technology naive or already using diabetes devices. The optimal CGM-based glycaemic goals and metrics for preventing in-hospital complications in the inpatient setting have yet to be fully explored.

## Conclusion

This position statement addresses the unmet need for clinical guidelines on use of CGM, non-AID insulin pumps and AID in non-critically ill hospitalised adults. The primary goal of this statement is to promote effective, safe and consistent use of these technologies to improve inpatient glycaemic management, enhance clinical outcomes and support the standardisation of the adoption of these technologies in hospital settings. Importantly, as these clinical recommendations are based on review of literature on adults, they cannot be directly extrapolated to the care of hospitalised paediatric populations.

## Supplementary Information

Below is the link to the electronic supplementary material.

Supplementary file1 (PDF 323 KB)

## Data Availability

No new data were created or analysed in this article. All data supporting the findings of this consensus report are available within the article and its electronic supplementary material, or from the references cited within the article.

## Abbreviations

ADA: American Diabetes Association
AID: Automated insulin delivery
CGM: Continuous glucose monitoring
CT: Computed tomography
EASD: European Association for the Study of Diabetes
EHR: Electronic health records
FDA: Food and Drug Administration
MDI: Multiple daily injection
POC: Point-of-care
RCT: Randomised controlled trials

## References

[1] Olsen MT, Klarskov CK, Jensen SH et al (2025) In-hospital diabetes management by a diabetes team and insulin titration algorithms based on continuous glucose monitoring or point-of-care glucose testing in patients with type 2 diabetes (DIATEC): a randomized controlled trial. Diabetes Care 48:569–578. 10.2337/dc24-2222 39887698

[2] Spanakis EK, Urrutia A, Galindo RJ et al (2022) Continuous glucose monitoring-guided insulin administration in hospitalized patients with diabetes: a randomized clinical trial. Diabetes Care 45:2369–2375. 10.2337/dc22-0716 PMC9643134 35984478

[3] Singh LG, Satyarengga M, Marcano I et al (2020) Reducing inpatient hypoglycemia in the general wards using real-time continuous glucose monitoring: the glucose telemetry system, a randomized clinical trial. Diabetes Care 43:2736–2743. 10.2337/dc20-0840 PMC7576426 32759361

[4] Lima Chagas GC, Teixeira L, Clemente MRC et al (2025) Use of continuous glucose monitoring and point-of-care glucose testing in hospitalized patients with diabetes mellitus in non-intensive care unit settings: a systematic review and meta-analysis of randomized controlled trials. Diabetes Res Clin Pract 220:111986. 10.1016/j.diabres.2024.111986 39798897

[5] Olsen MT, Jensen SH, Rasmussen LM et al (2025) Glycemic levels and insulin use daily and during hospital shifts in hospitalized patients with type 2 diabetes. J Endocr Soc 9:bvaf137. 10.1210/jendso/bvaf137 PMC12448850 40980547

[6] Umpierrez GE, Isaacs SD, Bazargan N, You X, Thaler LM, Kitabchi AE (2002) Hyperglycemia: an independent marker of in-hospital mortality in patients with undiagnosed diabetes. J Clin Endocrinol Metab 87:978–982. 10.1210/jcem.87.3.8341 11889147

[7] Olsen MT, Dungu AM, Klarskov CK, Jensen AK, Lindegaard B, Kristensen PL (2022) Glycemic variability assessed by continuous glucose monitoring in hospitalized patients with community-acquired pneumonia. BMC Pulm Med 22:83. 10.1186/s12890-022-01874-7 PMC8905566 35264139

[8] Baker EH, Janaway CH, Philips BJ et al (2006) Hyperglycaemia is associated with poor outcomes in patients admitted to hospital with acute exacerbations of chronic obstructive pulmonary disease. Thorax 61:284–289. 10.1136/thx.2005.051029 PMC2104606 16449265

[9] Simó-Servat O, Amigó J, Ortiz-Zúñiga Á et al (2025) Smart diabetes hospital: clinical impact in complex surgical units of a tertiary hospital. Acta Diabetol 62:423–428. 10.1007/s00592-024-02370-6 PMC11872769 39240308

[10] Barmanray RD, Kyi M, Colman PG et al (2024) The Specialist Treatment of Inpatients: Caring for Diabetes in Surgery (STOIC-D Surgery) trial: a randomized controlled trial of early intervention with an electronic specialist-led model of diabetes care. Diabetes Care 47:948–955. 10.2337/dc23-1905 38237121

[11] Umpierrez GE, Klonoff DC (2018) Diabetes technology update: use of insulin pumps and continuous glucose monitoring in the hospital. Diabetes Care 41:1579–1589. 10.2337/dci18-0002 PMC6054505 29936424

[12] Avari P, Lumb A, Flanagan D et al (2023) Continuous glucose monitoring within hospital: a scoping review and summary of guidelines from the Joint British Diabetes Societies for Inpatient Care. J Diabetes Sci Technol 17:611–624. 10.1177/19322968221137338 PMC10210120 36444418

[13] Lumb A, Misra S, Rayman G et al (2023) Variation in the current use of technology to support diabetes management in UK hospitals: results of a survey of health care professionals. J Diabetes Sci Technol 17:733–741. 10.1177/19322968231161076 PMC10210106 36949718

[14] Irace C, Coluzzi S, Di Cianni G et al (2023) Continuous glucose monitoring (CGM) in a non-ICU hospital setting: the patient’s journey. Nutr Metab Cardiovasc Dis 33:2107–2118. 10.1016/j.numecd.2023.06.021 37574433

[15] Akiboye F, Sihre HK, Al Mulhem M, Rayman G, Nirantharakumar K, Adderley NJ (2021) Impact of diabetes specialist nurses on inpatient care: a systematic review. Diabet Med 38:e14573. 10.1111/dme.14573 33783872

[16] Klarskov CK, Kristensen PL (2021) Experience from implementing telemetric in-hospital continuous glucose monitoring during the COVID-19 pandemic. J Diabetes Sci Technol 15:715–716. 10.1177/1932296821993169 PMC8120052 33593090

[17] Wallia A, Umpierrez GE, Rushakoff RJ et al.; DTS Continuous Glucose Monitoring in the Hospital Panel (2017) Consensus statement on inpatient use of continuous glucose monitoring. J Diabetes Sci Technol 11:1036–1044. 10.1177/1932296817706151 PMC5950996 28429611

[18] Peters AL, Ahmann AJ, Battelino T et al (2016) Diabetes technology-continuous subcutaneous insulin infusion therapy and continuous glucose monitoring in adults: an Endocrine Society clinical practice guideline. J Clin Endocrinol Metab 101:3922–3937. 10.1210/jc.2016-2534 27588440

[19] Spanakis EK, Cook CB, Kulasa K et al (2023) A consensus statement for continuous glucose monitoring metrics for inpatient clinical trials. J Diabetes Sci Technol 17:1527–1552. 10.1177/19322968231191104 PMC10658683 37592726

[20] American Diabetes Association Professional Practice Committee for Diabetes (2026) Introduction and methodology: Standards of Care in Diabetes—2026. Diabetes Care 49(Suppl. 1):S1–S5. 10.2337/dc26-SINT PMC12690168 41358883

[21] Lake A, Arthur A, Byrne C, Davenport K, Yamamoto JM, Murphy HR (2019) The effect of hypoglycaemia during hospital admission on health-related outcomes for people with diabetes: a systematic review and meta-analysis. Diabet Med 36:1349–1359. 10.1111/dme.14115 PMC7004204 31441089

[22] Galindo RJ, Umpierrez GE, Rushakoff RJ et al (2020) Continuous glucose monitors and automated insulin dosing systems in the hospital consensus guideline. J Diabetes Sci Technol 14:1035–1064. 10.1177/1932296820954163 PMC7645140 32985262

[23] Shaw JLV, Bannuru RR, Beach L et al (2024) Consensus considerations and good practice points for use of continuous glucose monitoring systems in hospital settings. Diabetes Care 47:2062–2075. 10.2337/dci24-0073 PMC12328255 39452893

[24] Wilson LM, Herzig SJ, Marcantonio ER et al (2025) Management of diabetes and hyperglycemia in the hospital: a systematic review of clinical practice guidelines. Diabetes Care 48:655–664. 10.2337/dc24-2510 PMC11932812 40117466

[25] Hirsch IB, Draznin B, Buse JB et al (2025) Results from a randomized trial of intensive glucose management using CGM versus usual care in hospitalized adults with type 2 diabetes: the TIGHT study. Diabetes Care 48:118–124. 10.2337/dc24-1779 PMC12328254 39571106

[26] Thabit H, Rubio J, Karuppan M et al (2024) Use of real-time continuous glucose monitoring in non-critical care insulin-treated inpatients under non-diabetes speciality teams in hospital: a pilot randomized controlled study. Diabetes Obes Metab 26:5483–5487. 10.1111/dom.15885 39169520

[27] Olsen MT, Vikner ME, Jensen SH et al (2025) Satisfaction with continuous glucose monitoring and diabetes care among hospitalised patients with type 2 diabetes managed by inpatient diabetes teams. Diabet Med 42:e70114. 10.1111/dme.70114 40722231

[28] Olsen MT, Liarakos AL, Wilmot EG et al (2025) Implementation strategies for inpatient continuous glucose monitoring-based diabetes management: a systematic review. J Clin Endocrinol Metab 110:e2411–e2419. 10.1210/clinem/dgaf074 39918053

[29] Philis-Tsimikas A, San Diego ERN, Vincent L, Lohnes S, Singleton C (2024) Are we ready for real-time continuous glucose monitoring in the hospital setting? Benefits, challenges, and practical approaches for implementation: case vignette: remote real-time continuous glucose monitoring for hospitalized care in Quincy Koala. Curr Diab Rep 24:217–226. 10.1007/s11892-024-01549-z PMC13441287 39126617

[30] Olsen MT, Jensen SH, Rasmussen LM et al (2025) Most hospitalised patients with type 2 diabetes benefit from continuous glucose monitoring compared to point-of-care glucose testing in a non-intensive care unit setting: a heterogeneity of treatment effect analysis. Diabetes Obes Metab 27:2857–2863. 10.1111/dom.16297 40000406

[31] Gu W, Liu Y, Chen Y et al (2017) Multicentre randomized controlled trial with sensor-augmented pump vs multiple daily injections in hospitalized patients with type 2 diabetes in China: time to reach target glucose. Diabetes Metab 43:359–363. 10.1016/j.diabet.2016.12.009 28236571

[32] Davis GM, Hughes MS, Brown SA et al (2023) automated insulin delivery with remote real-time continuous glucose monitoring for hospitalized patients with diabetes: a multicenter, single-arm, feasibility trial. Diabetes Technol Ther 25:677–688. 10.1089/dia.2023.0304 PMC10611957 37578778

[33] Boughton CK, Hartnell S, Hobday N et al (2023) Implementation of fully closed-loop insulin delivery for inpatients with diabetes: real-world outcomes. Diabet Med 40:e15092. 10.1111/dme.15092 36947090

[34] Pelkey MN, Boyle ME, Long A, Castro JC, Cook CB, Thompson B (2023) Hybrid closed-loop insulin pump technology can be safely used in the inpatient setting. Endocr Pract 29:24–28. 10.1016/j.eprac.2022.11.006 36400399

[35] Thompson B, Boyle ME, Castro JC, Dodoo C, Cook CB (2024) Automated insulin delivery technology in the hospital: update on safety and efficacy data. Endocr Pract 30:957–962. 10.1016/j.eprac.2024.07.012 39053588

[36] Thabit H, Hartnell S, Allen JM et al (2017) Closed-loop insulin delivery in inpatients with type 2 diabetes: a randomised, parallel-group trial. Lancet Diabetes Endocrinol 5:117–124. 10.1016/S2213-8587(16)30280-7 27836235

[37] Bally L, Thabit H, Hartnell S et al (2018) Closed-loop insulin delivery for glycemic control in noncritical care. N Engl J Med 379:547–556. 10.1056/NEJMoa1805233 29940126

[38] Boughton CK, Bally L, Martignoni F et al (2019) Fully closed-loop insulin delivery in inpatients receiving nutritional support: a two-centre, open-label, randomised controlled trial. Lancet Diabetes Endocrinol 7:368–377. 10.1016/S2213-8587(19)30061-0 PMC6467839 30935872

[39] Benhalima K, Polsky S (2026) Automated insulin delivery in pregnancies complicated by type 1 diabetes. J Diabetes Sci Technol 20:38–49. 10.1177/19322968251323614 PMC11904923 40071788

[40] Thabit H, Schofield J (2024) Technology in the management of diabetes in hospitalised adults. Diabetologia 67:2114–2128. 10.1007/s00125-024-06206-4 PMC11447115 38953925

[41] Thompson B, Korytkowski M, Klonoff DC, Cook CB (2018) Consensus statement on use of continuous subcutaneous insulin infusion therapy in the hospital. J Diabetes Sci Technol 12:880–889. 10.1177/1932296818769933 PMC6134295 29681173

[42] Olsen MT, Klarskov CK, Hansen KB, Pedersen-Bjergaard U, Kristensen PL (2024) Risk factors at admission of in-hospital dysglycemia, mortality, and readmissions in patients with type 2 diabetes and pneumonia. J Diabetes Complications 38:108803. 10.1016/j.jdiacomp.2024.108803 38959725

[43] Cruz P (2020) Inpatient hypoglycemia: the challenge remains. J Diabetes Sci Technol 14:560–566. 10.1177/1932296820918540 PMC7576945 32389071

[44] Pratiwi C, Mokoagow MI, Made Kshanti IA, Soewondo P (2020) The risk factors of inpatient hypoglycemia: a systematic review. Heliyon 6:e03913. 10.1016/j.heliyon.2020.e03913 PMC7218453 32420485

[45] Umpierrez GE, Holt EH, Einhorn D, McGill JB (2020) Concentrated insulins: clinical update of therapeutic options. Endocr Pract 26:1–12. 10.4158/EP-2019-0607 32339033

[46] Wei SW, Jiang SQ (2023) Risk factors for glycemic control in hospitalized patients with type 2 diabetes receiving continuous subcutaneous insulin infusion therapy. Diabetes Therapy 14:167–178. 10.1007/s13300-022-01342-9 PMC9880112 36454378

[47] Pasquel FJ, Umpierrez GE (2017) Management of inpatient hyperglycemia and diabetes in older adults. Diabetes Care 40:509–517. 10.2337/dc16-0989 PMC5864102 28325798

[48] American Diabetes Association Professional Practice Committee for Diabetes (2026) 13. Older adults: standards of care in diabetes—2026. Diabetes Care 49(Suppl 1):S277–S296. 10.2337/dc26-S013 PMC12690186 41358888

[49] Olsen MT, Nørgaard K, Jensen SH et al (2026) Acute kidney injury in hospitalised patients with type 2 diabetes: associations with continuous glucose monitoring-based glucose levels, mortality, and ICU admission. Diabet Med 43:e70321. 10.1111/dme.70321 41964006

[50] Avari P, Lumb A, Flanagan D et al (2023) Insulin pumps and hybrid close loop systems within hospital: a scoping review and practical guidance from the joint British diabetes societies for inpatient care. J Diabetes Sci Technol 17:625–634. 10.1177/19322968221137335 PMC10210119 36458697

[51] Fortmann AL, Bagsic SRS, Talavera L et al (2020) Glucose as the fifth vital sign: a randomized controlled trial of continuous glucose monitoring in a non-ICU hospital setting. Diabetes Care 43:2873–2877. 10.2337/dc20-1016 PMC7576427 32855160

[52] Haas NL, Ang L, Esfandiari NH et al (2025) Analytical accuracy of a continuous glucose monitor in adult diabetic ketoacidosis. CHEST Critical Care 3:100109. 10.1016/j.chstcc.2024.100109

[53] Battelino T, Danne T, Bergenstal RM et al (2019) Clinical targets for continuous glucose monitoring data interpretation: recommendations from the international consensus on time in range. Diabetes Care 42:1593–1603. 10.2337/dci19-0028 PMC6973648 31177185

[54] Olsen MT, Hansen KB, Pedersen-Bjergaard U, Kristensen PL (2025) Time at high risk of hypoglycemia – validation of a consensus-based continuous glucose monitoring-metric for hospitalized patients. J Diabetes Sci Technol. 10.1177/19322968251331628 PMC11993535 40219621

[55] Flory JH, Aleman JO, Furst J, Seley JJ (2014) Basal insulin use in the non-critical care setting. J Diabetes Sci Technol 8:427–428. 10.1177/1932296813520367 PMC4455403 24876599

[56] Olsen MT, Klarskov CK, Bjergaard UP, Hansen KB, Kristensen PL (2024) Summary of clinical investigation plan for The DIATEC trial: in-hospital diabetes management by a diabetes team and continuous glucose monitoring or point of care glucose testing – a randomised controlled trial. BMC Endocr Disord 24(1):60. 10.1186/s12902-024-01595-4 PMC11071255 38711112

[57] Olsen MT, Pedersen-Bjergaard U, Jensen SH, Rasmussen LM, Klarskov CK (2025) Evaluation of a continuous glucose monitoring-based insulin titration protocol for inpatients with type 2 diabetes in nonintensive care unit settings. J Diabetes Sci Technol 19322968251331628. 10.1177/19322968251331628 PMC11977621 40186499

[58] US Food and Drug Administration (2022) Medical devices; clinical chemistry and clinical toxicology devices; classification of the integrated continuous glucose monitoring system. 21 CFR Part 862. Fed Regist 87:9237–9239. www.federalregister.gov/documents/2022/02/18/2022-03504/medical-devices-clinical-chemistry-and-clinical-toxicology-devices-classification-of-the-integrated. Accessed 12 Jan 2026

[59] American Diabetes Association Professional Practice Committee for Diabetes (2025) 16. Diabetes care in the hospital: standards of care in diabetes—2025. Diabetes Care 48:S321–S334. 10.2337/dc25-S016 PMC11635037 39651972

[60] Putzu A, Grange E, Schorer R, Schiffer E, Gariani K (2025) Continuous peri-operative glucose monitoring in noncardiac surgery. Eur J Anaesthesiol 42:162–171. 10.1097/EJA.0000000000002095 PMC11676603 39512161

[61] Cruz P, McKee AM, Chiang H-H et al (2025) Perioperative care of patients using wearable diabetes devices. Anesth Analg 140:2–12. 10.1213/ANE.0000000000007115 38913575

[62] Martin LD, Hoagland MA, Rhodes ET, Wolfsdorf JI, Hamrick JL (2020) Perioperative management of pediatric patients with type 1 diabetes mellitus, updated recommendations for anesthesiologists. Anesth Analg 130:821–827. 10.1213/ANE.0000000000004491 31688079

[63] Sreedharan R, Khanna S, Shaw A (2023) Perioperative glycemic management in adults presenting for elective cardiac and non-cardiac surgery. Perioper Med 12:13. 10.1186/s13741-023-00302-6 PMC10149003 37120562

[64] Partridge H, Perkins B, Mathieu S, Nicholls A, Adeniji K (2016) Clinical recommendations in the management of the patient with type 1 diabetes on insulin pump therapy in the perioperative period: a primer for the anaesthetist. Br J Anaesth 116:18–26. 10.1093/bja/aev347 26675948

[65] Rajan N, Duggan EW, Abdelmalak BB et al (2024) Society for ambulatory anesthesia updated consensus statement on perioperative blood glucose management in adult patients with diabetes mellitus undergoing ambulatory surgery. Anesth Analg 139:459–477. 10.1213/ANE.0000000000006791 38517760

[66] Yeh T, Yeung M, Mendelsohn Curanaj FA (2021) Managing patients with insulin pumps and continuous glucose monitors in the hospital: to wear or not to wear. Curr Diab Rep 21:7. 10.1007/s11892-021-01375-7 PMC7810103 33449214

[67] Lal S, Khan E (2024) Perioperative management of diabetic patients: optimising care with insulin pumps and CGM devices. Available from: https://resources.wfsahq.org/wp-content/uploads/ATOTW_524.pdf. Accessed 12 Jan 2026

[68] Sobel SI, Augustine M, Donihi AC, Reider J, Forte P, Korytkowski M (2015) Safety and efficacy of a peri-operative protocol for patients with diabetes treated with continuous subcutaneous insulin infusion who are admitted for same-day surgery. Endocr Pract 21:1269–1276. 10.4158/EP15727.OR 26280203

[69] Corney SM, Dukatz T, Rosenblatt S et al (2012) Comparison of insulin pump therapy (Continuous Subcutaneous Insulin Infusion) to alternative methods for perioperative glycemic management in patients with planned postoperative admissions. J Diabetes Sci Technol 6:1003–1015. 10.1177/193229681200600503 PMC3570833 23063025

[70] Kannan S, Satra A, Calogeras E, Lock P, Lansang MC (2014) Insulin pump patient characteristics and glucose control in the hospitalized setting. J Diabetes Sci Technol 8:473–478. 10.1177/1932296814522809 PMC4455446 24876608

[71] Boyle ME, Seifert KM, Beer KA et al (2012) Insulin pump therapy in the perioperative period: a review of care after implementation of institutional guidelines. J Diabetes Sci Technol 6:1016–1021. 10.1177/193229681200600504 PMC3570834 23063026

[72] Mackey PA, Thompson BM, Boyle ME et al (2015) Update on a quality initiative to standardize perioperative care for continuous subcutaneous insulin infusion therapy. J Diabetes Sci Technol 9:1299–1306. 10.1177/1932296815592027 PMC4667318 26092687

[73] Long MT, Coursin DB, Rice MJ (2019) Perioperative considerations for evolving artificial pancreas devices. Anesth Analg 128:902–906. 10.1213/ANE.0000000000003779 30198923

[74] Herzig D, Suhner S, Roos J et al (2022) Perioperative fully closed-loop insulin delivery in patients undergoing elective surgery: an open-label, randomized controlled trial. Diabetes Care 45:2076–2083. 10.2337/dc22-0438 35880252

[75] Galindo RJ, Aleppo G, Klonoff DC et al (2020) Implementation of continuous glucose monitoring in the hospital- emergent considerations for remote glucose monitoring during the COVID-19 pandemic. J Diabetes Sci Technol 14:822–832. 10.1177/1932296820932903 PMC7673156 32536205

[76] Spanakis EK, Levitt DL, Siddiqui T et al (2018) The effect of continuous glucose monitoring in preventing inpatient hypoglycemia in general wards: the glucose telemetry system. J Diabetes Sci Technol 12:20–25. 10.1177/1932296817748964 PMC5761998 29237288

[77] Dillmann C, Amoura L, Fall Mostaine F, Coste A, Bounyar L, Kessler L (2022) Feasibility of real-time continuous glucose monitoring telemetry system in an inpatient diabetes unit: a pilot study. J Diabetes Sci Technol 16:955–961. 10.1177/1932296821994586 PMC9264424 33660531

[78] Fonseca VA, Grunberger G, Anhalt H et al (2016) Continuous glucose monitoring: a consensus Conference of the American Association of Clinical Endocrinologists and American College of Endocrinology. Endocr Pract 22:1008–1021. 10.4158/EP161392.CS 27214060

[79] Bellido V, Freckman G, Pérez A, Galindo RJ (2023) Accuracy and potential interferences of continuous glucose monitoring sensors in the hospital. Endocr Pract 29:919–927. 10.1016/j.eprac.2023.06.007 37369291

[80] Davis GM, Spanakis EK, Migdal AL et al (2021) Accuracy of dexcom G6 continuous glucose monitoring in non-critically ill hospitalized patients with diabetes. Diabetes Care 44:1641–1646. 10.2337/dc20-2856 PMC8323182 34099515

[81] Perez-Guzman MC, Duggan E, Gibanica S et al (2021) Continuous glucose monitoring in the operating room and cardiac intensive care unit. Diabetes Care 44:e50–e52. 10.2337/dc20-2386 PMC7896262 33479159

[82] Freckmann G, Pleus S, Grady M, Setford S, Levy B (2019) Measures of accuracy for continuous glucose monitoring and blood glucose monitoring devices. J Diabetes Sci Technol 13:575–583. 10.1177/1932296818812062 PMC6501529 30453761

[83] Avari P, Choudhary P, Lumb A et al (2025) Using technology to support diabetes care in hospital: guidelines from the Joint British Diabetes Societies for Inpatient Care (JBDS‐IP) group and Diabetes Technology Network (DTN) UK. Diabet Med 42. 10.1111/dme.15452 PMC11823341 39432570

[84] O’Connor MY, Flint KL, Sabean A et al (2024) Accuracy of continuous glucose monitoring in the hospital setting: an observational study. Diabetologia 67:2650–2659. 10.1007/s00125-024-06250-0 PMC12509055 39126488

[85] Flanagan D, Avari P, Choudhary P et al (2023) Using technology to improve diabetes care in hospital: the challenge and the opportunity. J Diabetes Sci Technol 17:503–508. 10.1177/19322968221138299 PMC10012371 36433805

[86] Xu NY, Nguyen KT, DuBord AY et al (2022) The launch of the iCoDE standard project. J Diabetes Sci Technol 16:887–895. 10.1177/19322968221093662 PMC9264445 35533135

[87] Aaron RE, Tian T, Yeung AM, Huang J, Klonoff DC, Espinoza JC (2024) The launch of the iCoDE-2 standard project: integration of connected diabetes device data into the electronic health record. J Diabetes Sci Technol 18:82–88. 10.1177/19322968231207888 PMC10899851 37850590

[88] Espinoza J, Klonoff D, Carini S (2025) 2025 iCoDE Report: EHR integration standards and recommendations for insulin delivery data. Available from: https://diabetestechnology.org/icode2. Accessed 12 Jan 2026

[89] Selmer C, Green A, Madsen S, Johannesen M, Jensen MT, Nørgaard K (2025) Stenopool: a comprehensive platform for consolidating diabetes device data. J Diabetes Sci Technol 19:1486–1495. 10.1177/19322968241264761 PMC11571449 39044480

[90] Matievich W, Kiaie N, Dunn TC (2023) Safety and functional integrity of continuous glucose monitoring sensors when used during radiologic procedures under high exposure conditions. J Diabetes Sci Technol 17:1634–1643. 10.1177/19322968221106206 PMC10658671 35771038

[91] Migdal AL, Spanakis EK, Galindo RJ et al (2020) Accuracy and precision of continuous glucose monitoring in hospitalized patients undergoing radiology procedures. J Diabetes Sci Technol 14:1135–1136. 10.1177/1932296820930038 PMC7645137 32476459

[92] Thomas C, Welsh JB, Lu S, Gray JM (2021) Safety and functional integrity of continuous glucose monitoring components after simulated radiologic procedures. J Diabetes Sci Technol 15:781–785. 10.1177/1932296820920948 PMC8252147 32319318

[93] Tellez S, Hornung L, Smith E et al (2024) Real-world accuracy of a continuous glucose monitoring system after radiologic exposure. Pediatr Diabetes 2024:2210509. 10.1155/2024/2210509 PMC12017230 40302953

[94] US Food and Drug Administration (2024) Preventing damage to wearable diabetes devices during imaging and radiation procedures. Available from: https://www.fda.gov/radiation-emitting-products/electronic-medical-devices-x-ray-imaging-and-radiation-therapy-what-know-and-how-prevent-damage/preventing-damage-wearable-diabetes-devices-during-imaging-and-radiation-procedures. Accessed 24 Mar 2025

[95] Abbott (2024) Abbott’s FreeStyle Libre® 2 and 3: first continuous glucose monitoring systems approved for use during medical imaging. Available from: https://abbott.mediaroom.com/press-releases?item=124675. 2024. https://abbott.mediaroom.com/press-releases?item=124675. Accessed 8 Mar 2025

[96] Abbott (2024) Can you wear a CGM during an MRI? If it’s ours, you can. Available from: https://www.abbott.com/corpnewsroom/diabetes-care/can-you-wear-a-cgm-during-an-mri--if-its-ours-you-can.html. Accessed 28 March

[97] Eversense (2019) Senseonics Eversense CGM sensor receives indication for MRI. Available from: https://www.senseonics.com/investor-relations/news-releases/2019/01-30-2019-002321416. Accessed 29 April 2025

[98] Lazar D, Tao M, Matievich W, Kastanos K, Dunn TC (2025) Accuracy and functionality of select continuous glucose monitoring systems are not impacted by implantable cardioverter defibrillator devices. J Diabetes Sci Technol 19:1385–1391. 10.1177/19322968241260038 PMC11571575 38907649

[99] Umpierrez GE, Reyes D, Smiley D et al (2014) Hospital discharge algorithm based on admission HbA1c for the management of patients with type 2 diabetes. Diabetes Care 37:2934–2939. 10.2337/dc14-0479 PMC4207201 25168125

[100] Tian T, Aaron RE, Seley JJ et al (2024) Use of continuous glucose monitors upon hospital discharge of people with diabetes: promise, barriers, and opportunity. J Diabetes Sci Technol 18:207–214. 10.1177/19322968231200847 PMC10899827 37784246

[101] Umpierrez GE, Castro-Revoredo I, Moazzami B et al (2025) Randomized study comparing continuous glucose monitoring and capillary glucose testing in patients with type 2 diabetes after hospital discharge. Endocr Pract 31:286–291. 10.1016/j.eprac.2024.11.018 PMC12233000 39694328

[102] Kantipudi S, Idrees T, Castro-Revoredo I et al (2025) Assessment of glycemic control using continuous glucose monitoring during hospitalization and after discharge for older adults. J Diabetes Sci Technol 19:1427–1428. 10.1177/19322968251332656 PMC11993542 40219823

[103] Charlson ME, Carrozzino D, Guidi J, Patierno C (2022) Charlson comorbidity index: a critical review of clinimetric properties. Psychother Psychosom 91:8–35. 10.1159/000521288 34991091

