# Supplementary material for: An international position statement on practical approaches for inpatient continuous glucose monitoring, insulin pumps and automated insulin delivery systems in adults
Source: Diabetologia. 2026 Sep 22;69(12):3409–24. doi: 10.1007/s00125-026-06838-8 (PMC13714031; doi:10.1007/s00125-026-06838-8)
Supplement: Supplementary file 1 [file 125_2026_6838_MOESM1_ESM.pdf]

Electronic Supplementary Material 1

Search strings

Continuous glucose monitoring

("Hospitalization"[Mesh] OR "inpatients"[MeSH Terms] OR "in-patient*"OR "inpatient*" OR "hospital admission*" OR "hospital stay*" OR "hospitalizing" OR "hospitalising" OR "ward patient*" OR "admitted patient*" OR "patient* in hospital*" OR "hospital setting" OR hospitalized OR hospitalised) AND (("continuous glucose monitor*"[Title/Abstract]) OR ("continuous glucose monitor*"[All Fields] OR "CGM"[All Fields] OR "Continuous Glucose Monitoring"[MeSH Terms] OR "continuous glucose monitor*"[All Fields]))

Automated insulin delivery and insulin pumps

("Hospitalization"[Mesh] OR "inpatients"[MeSH Terms] OR "in-patient*"OR "inpatient*" OR "hospital admission*" OR "hospital stay*" OR "hospitalizing" OR "hospitalising" OR "ward patient*" OR "admitted patient*" OR "patient* in hospital*" OR "hospital setting" OR hospitalized OR hospitalised) AND (Automated insulin delivery OR automated insulin dosing OR AID OR continuous subcutaneous insulin infusion OR CSII OR insulin pump OR hybrid closed-loop system OR closed-loop OR artificial pancreas OR closed-loop System OR closed-loop insulin delivery)

Electronic Supplementary Material 2

CGM-based insulin titration protocol for basal, prandial, and correctional insulin for hospitalised adult individuals with diabetes on multiple daily injections.

(Importantly, the CGM-based insulin titration protocol is validated in hospitalised individuals with type 2 diabetes only. No validated protocol exists for type 1 diabetes. However, because the recommended insulin protocol relies on percentage-based adjustments in insulin doses, it inherently accounts for individual insulin sensitivity and may be applicable in type 1 diabetes, although this remains to be confirmed.)

Basal, prandial, and correctional insulin are titrated daily from 24-hour retrospective CGM viewing. Daily assessment of glycaemic status may be based partially on a visual interpretation of CGM-glucose curves rather than raw data calculations until available software adequately adapts.

Rules when titrating insulin

To address the fact that glucose levels often fluctuate between different ranges in an inpatient setting, the protocol introduces two rules for prandial and basal insulin titration in prioritised order.

- *Rule of the lowest:* Prioritises low glucose levels when both low and high glucose levels occur during a titration period and above the percentage-based threshold. For example, titration rules triggered by a hypoglycaemic range of 3.9–5.5 mmol/L (70–99 mg/dL) take precedence over those triggered by a higher range of e.g. 10.1–15.0 mmol/L (181–270 mg/dL).
- *Rule of the extremes:* The extremes of glucose levels *all below* or *all above* or *within and above* or *within and below* the intended glucose level titration range of 5.6–7.8 mmol/L (100–140 mg/dL) are prioritised. For example, titration rules triggered by a hyperglycaemic range of 10.1–15.0 mmol/L (181–270 mg/dL) take precedence over those triggered by a range of 7.9–10.0 mmol/L (141–180 mg/dL), as the former represents a greater deviation – an "extreme" – from the intended titration range of 5.6–7.8 mmol/L (100–140 mg/dL).

Protocol for CGM-based titration of basal, prandial, and correctional insulin

**Insulin prescriptions during hospitalisation**
**Basal insulin**<br><br>Basal insulin (e.g., insulin glargine U100 with a 24-hour covering profile) is recommended to be given or rescheduled to lunch to allow same-day insulin titration decisions during the morning to take effect the same day. **Starting basal insulin dose is 0.25 IU/kg/day**, rounded to the nearest unit. Exceptions are:

- Starting basal insulin dose is 0.15 IU/kg/day for individuals continuing their usual-of-care outpatient glucose lowering agents.
- Starting basal insulin dose is 0.20 IU/kg/day in individuals aged >75 years and/or with an eGFR <60 ml/min/1.73 m^2 and/or body mass index (BMI) < 22.5 kg/m^2, and not continuing their usual-of-care outpatient glucose lowering agents.
- Starting basal insulin is continued on the usual outpatient dose in individuals treated with basal insulin at home.
- Individuals on once-weekly insulins can be continued with titration once-weekly during hospitalisation.[1]
- In perioperative or fasting individuals, basal insulin is paused on the day of surgery or during fasting and individuals are managed by insulin infusion per local guideline. The full basal insulin dose is continued the following days.

Prandial insulin

(ordered at the admission or at the discretion of the clinicals when glucose levels >10.0 mmol/L (>180 mg/dL) are observed postprandially for 1-2 days on the CGM)

Starting total prandial insulin dose is 0.25 IU/kg/day, rounded to the nearest unit, and given in three equally divided doses for breakfast, lunch, and dinner. Prandial insulin can also be given for selected meals only in the same standard doses per meal. Exceptions are:

- Starting prandial insulin dose is 0.15 IU/kg/day for individuals continuing their usual-of-care out-patient antidiabetic agents.
- Starting prandial insulin dose is 0.20 U/kg in individuals aged >75 years and/or with an eGFR <60 mL/min/1.73 m^2 and/or body mass index (BMI) <22.5 kg/m^2, and not continuing their usual-of-care out-patient antidiabetic agents.
- Prandial insulin is continued on the usual out-hospital dose in individuals treated with prandial insulin at home.
- When carbohydrate counting is feasible, an individual's usual insulin-to-carbohydrate ratio can be applied. Alternatively, if the carbohydrate content of the meal is predefined in the hospital setting but the individual's insulin-to-carbohydrate ratio is unknown, the "450 rule" may be used: dividing 450 by the total daily insulin dose yields the number of grams of carbohydrate covered by 1 unit of prandial insulin In perioperative or fasting individuals, prandial insulin is paused on the day of surgery or during fasting.
-

Correctional insulin

Correctional insulin is given by usual institutional protocol or an individual's known insulin sensitivity (correction) factor, at 03:00 h, preprandial at breakfast, lunch, and dinner, and before bedtime (22:00 h) or for individuals not eating at 03:00 h., 08:00 h., 12:00 h., 17:00 h., and 22:00 h and/or hyperglycaemic alarms for CGM-monitored individuals. Alternatively, the "100 rule" (or the 1800 rule when glucose is measured in mg/dL) may be used when measuring

glucose levels in mmol/L: dividing 100 (or 1800 for mg/dL) by the total daily insulin dose provides the estimated decrease in blood glucose per 1 unit of rapid-acting insulin, expressed in mmol/L or mg/dL, respectively.

Titrating basal, prandial, and correctional insulin

Titrating prandial insulin

Prandial insulin titration is guided by the nadir of the CGM glucose levels following the previous day's insulin administration. If no nadir is observed, the glucose level immediately before the next bolus guide titration.

Mmol/L	Mg/dL	Recommendation
<3.0	<54	Decrease insulin dose by 30%.
3.0–3.8	54–69	Decrease insulin dose by 20%.
3.9–5.5	70–99	Decrease insulin dose by 10%.
**5.6–7.8**	**100–140**	**No changes.**
7.9–10.0	141–180	Increase insulin dose by 10% if no hypoglycaemic events are observed the previous day
10.1–15.0	181–270	Increase insulin dose by 20% if no hypoglycaemic events were observed the previous day
>15.0	>270	Increase insulin dose by 30% if no hypoglycaemic events were observed the previous day

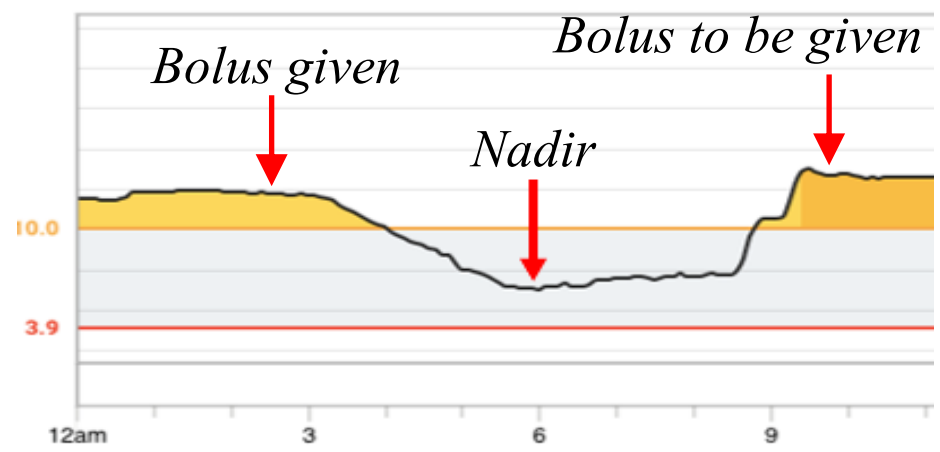

Titrating once daily basal insulin

Titration of **basal insulin** is done by glucose level ranges from 00:00 h till breakfast (07:00h).

Mmol/L	Mg/dL	Recommendation
<3.0	<54	for >5% (>20 min) of glucose levels and/or at least one hypoglycaemic event* < 3.0 mmol/L (< 54 mg/dL): Decrease dose by 30%
3.0–3.8	54–69	for >5% (>20 min) of glucose levels and/or at least one hypoglycaemic event* 3.0–3.8 mmol/L (54–69 mg/dL): Decrease dose by 20%
3.9–5.5	70–99	for >10% (>40 min) of glucose levels: Decrease dose by 10%
**5.6–7.8**	**100–140**	**No changes**
7.9–10.0	141–180	for >10% (>40 min) of glucose levels: Increase dose by 10%
10.1–15.0	181–270	for >10% (>40 min) of glucose levels: Increase dose by 20%
>15.0	>270	for >10% (>40 min) of glucose levels: Increase dose by 30%

* A CGM-detected hypoglycemic event is defined as three consecutive CGM glucose levels in the hypoglycemic range.[2]

References supplementary materials

(1) Philis-Tsimikas A, Krogsdahl Bache J, Fu A, Kellerer M, Salvesen-Sykes K, Bain SC. Insights on Hospitalisations from the Phase 3a ONWARDS 1–6 Trials of Once-Weekly Insulin Icodec. *Diabetes Therapy* 2025; published online June 4. DOI:10.1007/s13300-025-01745-4

(2) Battelino T, Alexander CM, Amiel SA, *et al.* Continuous glucose monitoring and metrics for clinical trials: an international consensus statement. *Lancet diabetes endocrinology* 2022; **11**: 42–57.
